# Proteomic Analysis Implicates Vimentin in Glioblastoma Cell Migration

**DOI:** 10.3390/cancers11040466

**Published:** 2019-04-03

**Authors:** Michal O. Nowicki, Josie L. Hayes, E. Antonio Chiocca, Sean E. Lawler

**Affiliations:** Harvey W. Cushing Neurooncology Laboratories, Department of Neurosurgery, Brigham and Women’s Hospital, Harvard Medical School, Boston, MA 02115, USA; mnowicki@bwh.harvard.edu (M.O.N.); jlhayes1982@gmail.com (J.L.H.); eachiocca@bwh.harvard.edu (E.A.C.)

**Keywords:** vimentin, GSK-3, glioblastoma, motility, cytoskeleton

## Abstract

We previously showed lithium chloride (LiCl) and other inhibitors of glycogen synthase kinase-3 (GSK-3) including 6-bromo-indirubin-3-oxime (BIO), can block glioblastoma (GBM) cell migration. To investigate the mechanisms involved we used two-dimensional difference in-gel electrophoresis (2D-DIGE) and mass spectrometry to identify proteins altered after treatment of U251 GBM cells with 20 mM LiCl. Downregulation of the intermediate filament protein vimentin was the most significant change identified. Analysis of patient tumor samples revealed that vimentin is expressed abundantly in GBM, and is prognostic especially in lower grade tumors. Additionally, siRNA-mediated vimentin knockdown impaired GBM migration. Western blotting showed that treatment with LiCl or small molecule GSK-3 inhibitors led to the rapid downregulation of detergent soluble vimentin levels across a panel of GBM-derived cells. Fluorescence reactivation after photobleaching (FRAP) microscopy studies showed a significant reduction in the ability of the vimentin cytoskeleton to recover from photo-bleaching in the presence of LiCl or BIO. Biochemical studies revealed that GSK-3 and vimentin directly interact, and analysis of vimentin revealed a GSK-3 consensus phosphorylation site. We conclude that anti-migratory compounds with the ability to inhibit GSK-3 have effects on vimentin cytoskeletal dynamics, which may play a role in their anti-invasive activity.

## 1. Introduction

Invasiveness is one of the main hallmarks of glioblastoma (GBM) and other glial tumors, in which malignant cells diffusely infiltrate normal brain tissue and migrate along blood vessels and defined structures of the brain [1,2]. This has important clinical consequences as it prevents complete surgical tumor resection, contributes to therapeutic resistance and helps to drive rapid tumor growth. Invasion is facilitated by multiple cellular processes including extracellular matrix remodeling [3], interactions of malignant cells with the normal cells of the brain and the host immune system [4], changes in cell adhesion properties [5] and cytoskeletal dynamics [6,7].

GBM is the most common malignant brain tumor and has a dismal prognosis [8,9]. At present there are no clinical approaches to block GBM invasion. We previously showed that lithium chloride (LiCl) potently and specifically blocks GBM cell migration in vitro [8]. LiCl has multiple targets within the cell [9,10,11], including the protein kinase glycogen synthase kinase-3 (GSK-3). GSK-3 is ubiquitously expressed in mammalian cells, with two closely related isoforms, GSK-3α and GSK-3β which have distinct and overlapping functions [12,13,14]. GSK-3 is a multi-tasking enzyme, known for its role in the regulation of glycogen synthesis, via inactivation by Akt, and also for its role as a critical mediator of Wnt signaling [15]. GSK-3 has been associated with a number of processes involved in cell motility via interactions with cytoskeletal components [11,16].

In pre-clinical murine GBM xenograft models we showed that protein kinase inhibitors of the indirubin family, which are known to potently inhibit GSK-3, block invasion and angiogenesis improving survival [17,18]. However, we have not yet defined the mechanisms underlying our observations in GBM cells. Here, we performed a proteomic study, which revealed consistent and highly significant downregulation of the intermediate protein vimentin after treatment of GBM cells with LiCl, and the indirubin derivative 6-bromo-indirubin-3-oxime (BIO).

Vimentin is a major component of the intermediate filament cytoskeleton and is best known in cancer as a marker of cellular epithelial to mesenchymal transition (EMT) [9], a phenomenon associated with cancer cell invasion and metastatic tumor spread. Vimentin is known to play an important role in maintenance of cellular integrity and resistance to stress, and vimentin knockout mice show impaired wound healing ability [19]. Vimentin has been shown to regulate cell migration via interaction with paxillin, integrin α6β4 and myosin II [20]. The role of vimentin in GBM has not been explored in detail regardless of the fact that it has been used as biomarker [21].

Here we show that LiCl and BIO, which block GBM cell migration, both affect the dynamic regulation of vimentin in GBM cells, and that vimentin knockdown partially blocks GBM migration. We also show that vimentin and GSK-3 physically associate. Thus, vimentin may affect GBM invasion downstream of anti-invasive compounds LiCl and indirubin derivatives.

## 2. Results

### 2.1. Two-Dimensional Fluorescence DIGE Proteomics Analysis Identifies Vimentin Downregulation by LiCl in GBM Cells

**Figure 1:**
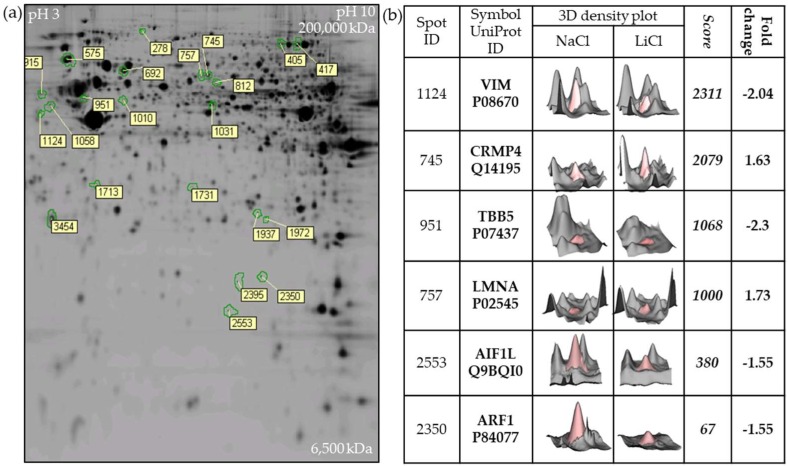
Two-dimensional fluorescence difference gel electrophoresis (DIGE) proteomics analysis identified 22 protein spots significantly altered by 20 mM lithium chloride (LiCl) treatment of glioblastoma (GBM) cells for 24 h. The experiment was performed in triplicate. (**a**) Representative Cy3/Cy5 stained (presented in gray) gel showing differentially regulated spots after 24 h treatment of U251 cells with 20 mM LiCl. (**b**) The top six targets were excised from the gel based on significance after analysis of alterations in fluorescence intensity post-LiCl treatment and identified by mass spectrometry peptide analysis.

Our previous studies showed that LiCl specifically and reversibly blocks GBM cell migration with minimal cytotoxicity in a dose-dependent manner with maximal effects at a concentration of 20 mM [8]. To better understand these observations, proteomic analysis was performed on U251 GBM cells treated with 20 mM LiCl. Image analysis after 2-D fluorescence difference gel electrophoresis (DIGE) revealed 22 significantly altered spots on the gel comparing LiCl treated U251 cells with untreated controls (≥1.5 fold average increase/decrease and *p* ≤ 0.05, Students *t*-test) (Figure 1a). Individual spots were cut from the gels, and mass spectrometry was used to determine the identity of the most significant hits identified by 2D-DIGE (Figure 1b). Interestingly, the six top primary targets identified (LMNA, ARF1, AIF1L, VIM, TUBB and CRMP4) are all components of the cytoskeleton, with the intermediate filament VIM (vimentin) being the top scoring hit. However, the role of intermediate filaments in GBM is poorly characterized. In this study, we therefore further explored the potential link between LiCl and vimentin in GBM as suggested by the proteomic data.

### 2.2. Vimentin Is Highly Expressed in GBM Patient Tumor Specimens and Is Associated with Cell Migration

**Figure 2:**
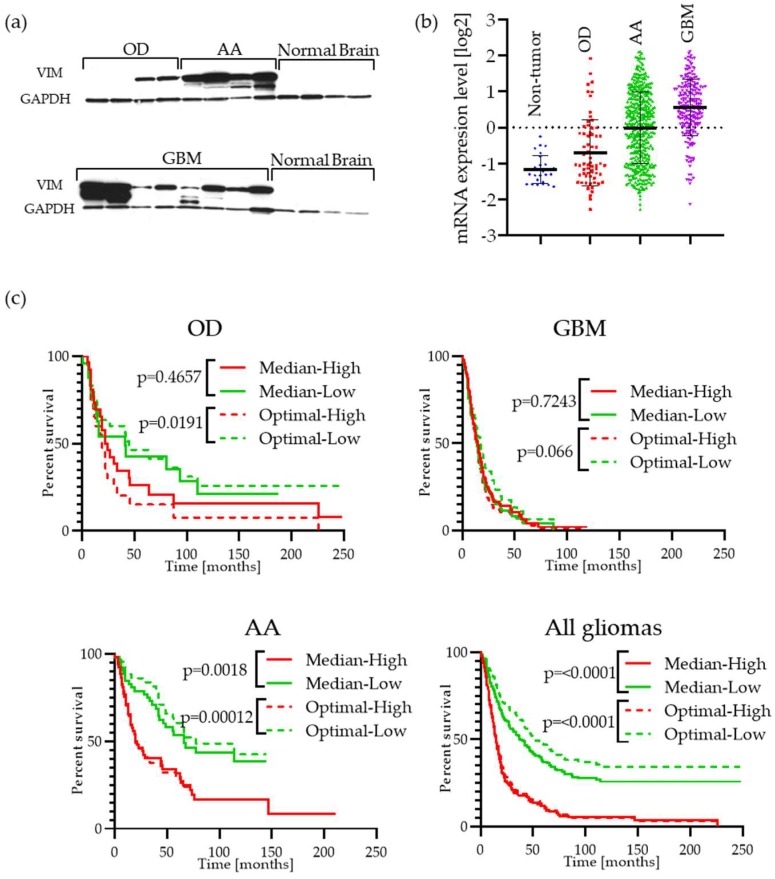
Expression of vimentin in malignant gliomas: (**a**) Western blot analysis of vimentin expression in human patient malignant glioma surgical specimens: OD—oligodendroglioma, AA—anaplastic astrocytoma, GBM—glioblastoma multiforme; compared with normal brain; (**b**) the mRNA transcript levels of vimentin according REMBRANDT database (http://www.betastasis.com/glioma/rembrandt), sample size: non-tumor *n* = 29, OD *n* = 67, AA *n* = 460, GBM *n* = 220; (**c**) survival curves based on high versus low vimentin mRNA expression obtained from the REMBRANDT database. The solid lines present data for samples with levels higher (red) or lower (green) than median of dataset; the dashed lines presents data for samples divided into two groups (higher—red or lower—green) based on the “optimal cut-off” algorithm [22].

Initially, to confirm the relevance of vimentin in GBM, we examined its expression by Western blotting of patient tumor specimens. This revealed high levels of vimentin in GBM, anaplastic astrocytoma (AA) and oligodendroglioma (OD) specimens compared with normal brain controls (Figure 2a). This was confirmed by examination of gene expression data which showed significantly elevated median vimentin expression in GBM (*p* = 3.5 × 10^−23^) compared with normal brain (Figure 2b) and also that vimentin expression is a prognostic factor in glioma analyzed in the Rembrandt glioma database (Figure 2c). Analysis of vimentin mRNA transcript levels in the TCGA database shows that it is present in all types of malignancies, where most invasive cancer types like melanoma and glioma show the highest levels (Figure S1a). The TCGA database allows for survival analysis of combined GBM and Low-Grade Glioma (LGG) datasets, as shown in Figure S1b; this data also suggest that vimentin is a prognostic factor in both GBM and LGG.

### 2.3. Vimentin Immunofluorescence Staining in GBM cells and in Patient Specimens

**Figure 3:**
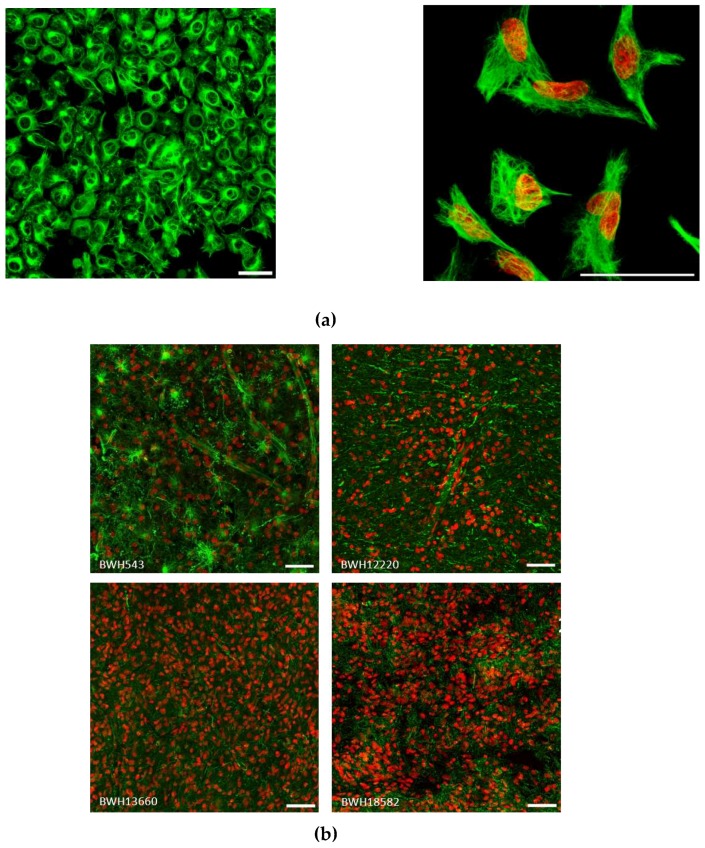
Vimentin staining in human GBM: (**a**) U251 cells grown as monolayers (left panel, field of view 400 by 400 μm, scale 50 µm) and high magnification (right, scale bar = 50 µm) of single cells, vimentin staining is presented here as a high dynamic range (HDR) image showing the perinuclear vimentin-rich region and fine network of filaments. (**b**) Surgically obtained human tumor specimens were stained for vimentin, field of view 400 by 400 µm, scale 50 µm.

To further understand vimentin in GBM we performed immunofluorescence staining in U251 GBM cells grown as monolayers. The vimentin cytoskeleton could clearly be seen ramifying throughout the cytosol, with a concentrated perinuclear area associated with the aggresome as previously observed [23] (Figure 3a). In patient specimens variable staining was observed, but in general a very strong signal could be seen throughout the tumor (Figure 3b). The small subset of glioma tissues in the Human Protein Atlas (www.proteinatlas.org) [24] are shown in Figure S2.

### 2.4. Vimentin Knockdown Reduces GBM Cell Migration

**Figure 4:**
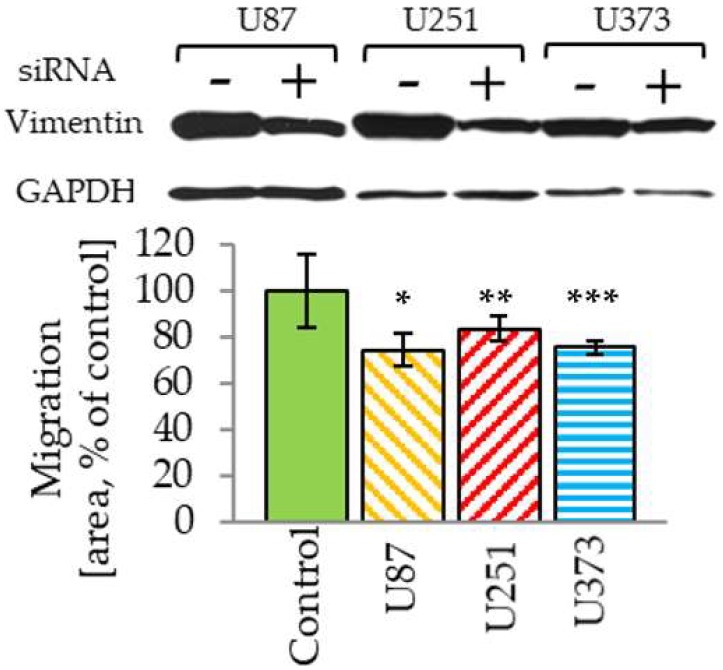
siRNA vimentin downregulation reduces the ability of cells to migrate across a transwell membrane in the modified Boyden chamber assay at 24 h post-transfection. The number of cells measured on the bottom side of membrane was normalized to the performance of the respective scrambled siRNA control (=100%) for each cell line; * *p* = 0.010, ** *p* = 0.015, *** *p* = 0.037. Western blot (upper) shows the actual degree of vimentin downregulation obtained for each cell line compared with scrambled siRNA control.

To establish further the relevance of vimentin in the process of GBM migration we performed siRNA knockdown. Even though we were not able to fully knockdown endogenous vimentin, similar to other reports [18,20], a significant reduction in transwell migration was observed in three distinct GBM cell lines (Figure 4).

### 2.5. Downregulation of the Triton-x-100 Soluble Vimentin Fraction in GBM Cell Lines by GSK-3 Inhibitors

**Figure 5:**
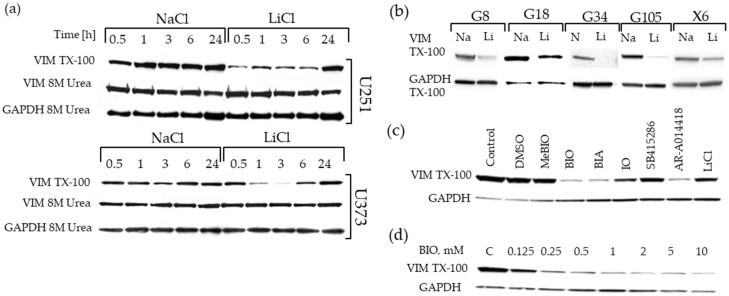
Downregulation of vimentin in detergent soluble extracts from GBM cells treated with LiCl, and a panel of small molecular glycogen synthase kinase-3 (GSK-3) inhibitors: (**a**) Validation in GBM cell lines. U251 and U373 glioma cells were treated with 20 mM LiCl or NaCl, for the time (hours) as indicated. Vimentin levels were determined in triton-x-100 extracts and in “total” extracts in 8 M urea. (**b**) Effects of 20 mM LiCl across a panel of GBM cell lines 3 h post-treatment. (**c**) Effects of a panel of GSK-3 inhibitors on vimentin levels in U251 cells 3 h post-treatment. (**d**) Dose-dependent effects of 6-bromo-indirubin-3-oxime (BIO) on triton-x-100 soluble vimentin levels 3 h post-treatment.

Having established the potential role of vimentin in GBM cell migration, we further investigated the effects of LiCl on vimentin levels in a range of GBM cell lines. Initially we used Western blotting to validate alterations in vimentin levels observed in the proteomics study. This confirmed the effects seen in our proteomics screen, with downregulation of vimentin expression observed in both U251 and U373 GBM cell lines, and was detected very rapidly, minutes after LiCl administration (Figure 5a). The effects were much more pronounced at earlier time points, with approximately 3-fold downregulation typically observed three hours post LiCl treatment. This effect was also observed in a panel of patient-derived GBM stem-like cells (GSCs) grown as neurospheres and treated with 20 mM LiCl for three hours (Figure 5b). The reduction of vimentin levels in the detergent soluble fraction was also observed in correlation with concentration dependent inhibition of GSK-3 by the indirubin derivative, 6-Bromoindirubin-3′-oxime (BIO) and other GSK-3 inhibitors (Figure 5c). The effect of BIO on vimentin levels was dose dependent (Figure 5d). These data suggest that vimentin downregulation is a common feature of these anti-invasive drugs. However, in all of these experiments lysates created in parallel using 8 M urea to solubilize all intracellular proteins did not show a difference in vimentin levels (shown in Figure 5a). Thus, there was in fact not a change in absolute vimentin levels, rather a change in its physical state, likely polymerized versus depolymerized within the cell. These data therefore suggest a change in the distribution of vimentin between intracellular triton-x-100 soluble and insoluble pools after treatment with GSK-3 inhibitors including LiCl and BIO.

### 2.6. Changes in Vimentin Dynamics in LiCl and BIO Treated GBM cells

**Figure 6:**
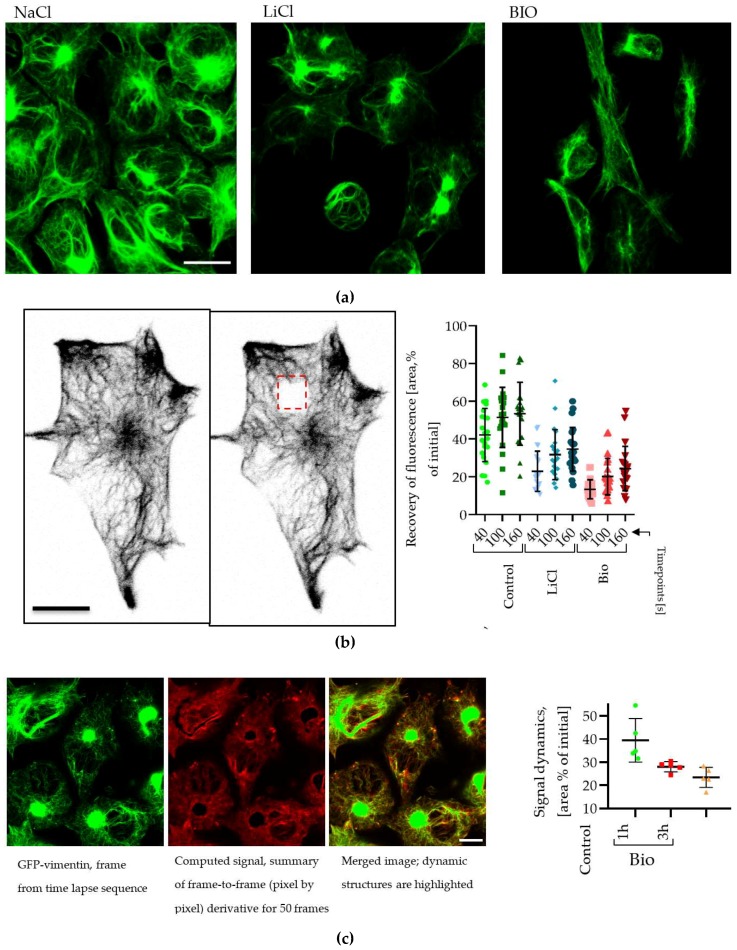
(**a**) Appearance of green fluorescent protein (GFP)-vimentin filaments after 20 mM LiCl or 5 μM BIO treatment (3 h, scale bar = 10 μm). (**b**) Recovery of photo-bleached GFP-vimentin in time points, scale bar = 10 μm. (**c**) Quantification of dynamic of vimentin skeleton movement by frame-to-frame derivative (μm^2^) in 50 frames (200 s) time lapse series; scale = 10 μm.

We used microscopic approaches to investigate these observations further using U251 cells stably expressing a green fluorescent protein (GFP)-vimentin fusion protein to visualize the effects of LiCl on the vimentin intermediate filament network. Exposure to 20 mM LiCl or 5 µM BIO for 3 h induced a reduction in the amount and GFP-intensity of small size vimentin fibers located at the cell periphery, and increased the GFP signal in the perinuclear area (Figure 6a). Time-lapse observations of GFP-vimentin fiber movement showed that in our cellular model, fibers are highly mobile laterally and possibly also have a dynamic rebuilding rate. We therefore utilized fluorescence recovery after photobleaching (FRAP) in which real-time fluorescence microscopy is used to determine the time taken for a cell to recover fluorescence levels after bleaching a small area in the presence and absence of drug treatment. A photobleached area was made in GFP-vimentin expressing U251 cells (Figure 6b), and recovery-time was measured. This showed a significant reduction in the time necessary for fibers to recover their fluorescent signal after LiCl or BIO treatment (Figure 6b, plot). We also utilized a time-lapse based approach to further assess effects of BIO treatment on fine vimentin network movement, which leads to up to 40% reduction in measured values compared with controls (Figure 6c). We also verified our previously reported observations [8,17] that LiCl and BIO treatments lead to overall reduction in cell motility in all tested cell lines and assays (Figure S3).

### 2.7. GSK-3 and Vimentin Colocalize in GBM Cells

**Figure 7:**
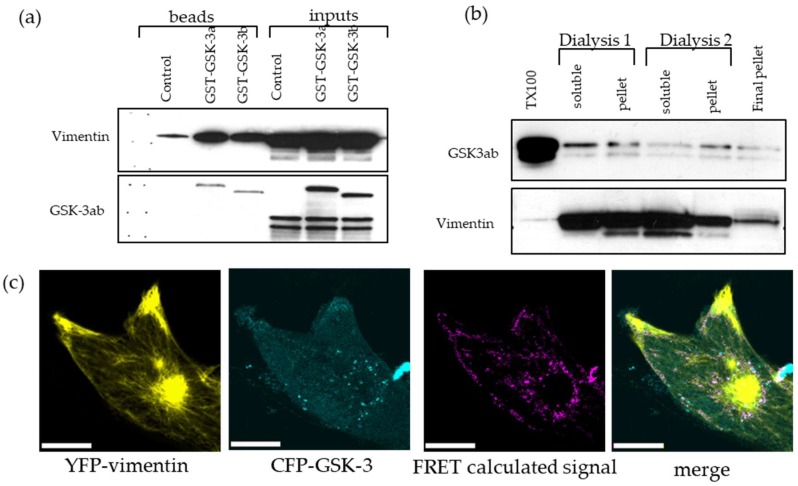
GSK-3 interaction with vimentin shown by: (**a**) glutathione-s-transferase (GST)-GSK-3 pull down assay; and (**b**) co-precipitation assay. GSK3 migrates as a double band with GSK-3α showing a slightly higher molecular weight than GSK-3β. (**c**) Yellow-fluorescent protein (YFP)-vimentin and cyan-fluorescent protein (CFP)-GSK-3β fusion proteins interaction as shown by Fluorescence REsonance Energy Transfer (FRET) colocalization, scale bar = 10 μm.

Both LiCl and BIO are known to inhibit the protein kinase GSK-3. Therefore, to investigate the relationship of vimentin and GSK-3, co-precipitation studies were performed. We used U251 cells transiently overexpressing glutathione-s-transferase (GST)-GSK-3α and GSK-3β fusion proteins. The cell lysate was incubated with agarose-glutathione beads, washed and subsequently assayed by Western blotting. We found that both isoforms of GSK-3 are able to interact with vimentin isolated from triton-x-100 cell lysates (Figure 7a). We then used a co-sedimentation assay to further investigate GSK-3 interaction with vimentin. In this assay, vimentin is solubilized and precipitated by a series of buffer exchanges in two cycles of dialysis, followed by Western blotting (Figure 7b). Additionally, Fluorescence Resonance Energy Transfer (FRET) measurements showed co-localization of cyan-fluorescent protein (CFP)-GSK3 and yellow-fluorescent protein (YFP)-vimentin fusion proteins in live cells (Figure 7c). Thus GSK-3 and vimentin are physically associated in GBM cells. These data identify a novel relationship between GSK-3 and vimentin for the first time and suggest that alterations in vimentin dynamics may contribute to the inhibition of GBM cell migration in response to LiCl and BIO.

## 3. Discussion

Our previous studies implicated the conserved, ubiquitous and multi-tasking protein kinase GSK-3 in GBM cell migration [8,17]. Here we performed a proteomics screen to investigate the mechanisms underlying these observations, and show for the first time that GSK-3 plays a role in regulation of the intermediate cytoskeleton component vimentin. Specifically, we establish that (1) GSK-3 regulates vimentin dynamics, (2) GSK-3 interacts with and phosphorylates vimentin, (3) vimentin facilitates GBM cell migration, and (4) vimentin is highly expressed in GBM patient specimens, and is prognostic, particularly in lower grade gliomas.

This study was performed as a development from our previously published observations on small molecules with potent inhibitory effects on GBM cell migration [8,17]. First, we showed that LiCl was a potent and reversible inhibitor of GBM cell migration in vitro [8]. These effects were observed at 10–20 mM LiCl, which exceeds the maximum tolerated dose of around 1 mM. LiCl is known to inhibit GSK-3 [25], therefore we went on to show that GSK-3α/β siRNA could block GBM migration and that other small molecule GSK-3 inhibitors could block GBM cell migration. During these studies, we examined derivatives of the Chinese medicine indirubin, which are known to inhibit GSK-3 [26]. These molecules blocked migration and angiogenesis in vivo in murine GBM models, improving animal survival [17].

To further study the underlying mechanisms, here we examined the impact of LiCl on GBM cells using proteomics-based approach. 2D-gel electrophoresis/mass spectrometry identified several proteins significantly altered by LiCl treatment. Interestingly these were all involved with cytoskeleton regulation and merit further study in the context of GBM cell migration. Indeed, one of these proteins, CRMP-4, regulates microtubule dynamics in axon outgrowth, and is also a known GSK-3 substrate [14]. Vimentin was the most significantly altered protein and the focus of this study. Additionally, by analysis of patient specimens by Western blotting and immunofluorescence, as well gene expression databases and the human protein atlas, we showed that vimentin is highly abundant in most GBM cases. At the mRNA level it is significantly prognostic in GBM and even more so in astrocytoma where the distribution of vimentin expression appears much broader than in GBM. Further studies are needed to determine whether the reduced expression in astrocytoma compared with GBM is at the tumor cell level, or rather reflects lower cellularity in lower grade tumors. To determine whether vimentin is truly a useful prognostic marker, much more detailed studies are required with larger well-characterized patient populations.

Our data suggest that a primary target of the anti-invasive drugs is vimentin dynamics. This is supported by the observations that (1) detergent soluble vimentin levels are rapidly reduced upon exposure to either LiCl or BIO, and (2) FRAP studies which showed a reduction in the ability of the vimentin cytoskeleton to recover after photobleaching of a GFP-vimentin fusion protein. Presumably this prevents the cell undergoing the restructuring and dynamic alterations required for effective migration and invasion.

Structurally, vimentin is a 57 kDa protein with a highly conserved α−helical rod domain flanked by N- and C-terminal domains which associate to ultimately form intermediate filament networks. Phosphorylation of vimentin, largely at the N-terminus, regulates its ability to form filaments and several kinases have been implicated in this process including protein kinase A (PKA), aurora kinase B and Akt which phosphorylate vimentin at various N-terminal sites [11,12]. Our data strongly implicates GSK-3 as a candidate vimentin kinase, although we have not yet definitively shown this. Analysis of the vimentin amino acid sequence reveals the presence of GSK-3 phosphorylation sites (Ser 50) [14,27] at the N-terminus, which are under investigation.

Further studies on the role of vimentin in GBM are warranted. This protein is highly abundant in GBM and is involved in multiple cellular processes including cell migration. However, one challenge we faced throughout this study was that vimentin manipulation in GBM cells is difficult; sustained knockdown was not straightforward due to the high levels of vimentin in GBM cells, and we have so far been unable to generate CRISPR gene edited GBM cell lines null for vimentin. Additionally, further detailed molecular studies are needed to understand better the effects of vimentin on GBM cell migration. Our data also shows that proteomics can be a very effective approach to identifying molecular changes relevant in GBM cell migration, revealing new targets and mechanisms.

## 4. Materials and Methods

### 4.1. Cell Lines, Tissue Samples and Chemicals

The U87 cell line was provided by Dr Webster Cavenee (Ludwig Institute for Cancer Research, La Jolla, CA, USA). U251 and U373 cells were purchased from ATCC (Manassas, VA, USA). All cells were grown in Dulbecco’s Modified Eagles Medium (DMEM) with 10% FBS and 1% penicillin-streptomycin. The GBM stem-like cells (GSCs) were derived from fresh samples of surgically resected brain tumors obtained from The Ohio State University Tissue Procurement Shared Resources under a protocol approved by OSU IRB. GSCs were maintained as neurosphere suspension cultures in Neurobasal medium with 20 ng/mL fibroblast growth factor (FGF) and epidermal growth factor (EGF). Unless otherwise stated, reagents, chemicals and supplies were purchased from Thermo-Fisher Scientific (Waltham, MA, USA).

### 4.2. Western Blotting

Proteins from 5 × 10^6^ cells were isolated by 15 min extraction in TX100 lysis buffer: 1% Triton X100, 50 mM Tris pH 7.5, 100 mM NaCl, 1 mM EDTA, 1 mM EGTA, 50 mM β-glycerophosphate supplemented with 50 mM NaF, 1 mM PMSF, 1 mM DTT, 2 mM Na_3_VO_4_, 10 μg/mL Leupeptin, 10 µg/mL Aprotinin, 10 μg/mL Pepstatin, 0.25 ng/mL Microcystin LR (EMD Millipore, Billerica, MA, USA). To achieve total solubilization of vimentin fibers, TX100 lysis buffer was supplemented with 8 M urea. Tissue samples were solubilized by sonication in urea containing TX100 lysis buffer. For Western blotting, proteins (100 μg) were separated by SDS-PAGE (Criterion system, Bio-Rad, Hercules, CA, USA) and transferred to nitrocellulose (Bio-Rad). Antibodies used for Western blotting were: mouse anti-vimentin V9 (MS-129-P, Thermo Scientific, (Waltham, MA, USA) mouse anti-GSK-3αβ (368662, EMD Millipore), mouse anti-α-tubulin (T6074, Sigma-Aldrich, St. Louis, MO, USA), mouse anti-GAPDH (ab9484-200, Abcam, Cambridge, MA, USA) and peroxidase-conjugated secondary antibodies (Jackson Laboratories, Bar Harbor, ME, USA).

### 4.3. Cell Migration Assays

For vimentin knockdown, 200 pmols of Smartpool siRNA, or scrambled siRNA control (Dharmacon, Chicago, IL, USA) was introduced into cells with Lipofectamine 2000 (Thermo Scientific) in a six-well plate 24 h prior to the transwell assay (modified Boyden chamber assay [28]). Assays were performed using 8 μm pore size inserts (ISC Bioexpress, Kaysville, UT, USA), and 50,000 cells per well. Cells were allowed to migrate for 6 h prior to fixation in 1% glutaraldehyde and cell visualization was performed by staining with 10 µg/mL 4′,6-diamidino-2-phenylindole (DAPI). Images of the whole membrane were captured with a motorized Nikon Eclipse Ti microscope system. The time-lapse version of the transwell assay was performed with FluoroBlock inserts (Corning cat# 351152 & 353504) and U251pCDH (copGFP labeled cells, System Biosciences plasmid pCDH-EF1-MCS-T2A-copGFP). Images of the whole insert were captured with a motorized Nikon Eclipse TE2000 microscope system (Nikon, Tokyo, Japan) equipped with an on-stage incubator with CO_2_ and temperature control. The invasion/migration assay performed in collagen gel was performed as described previously [8]. Analysis and quantification of signal was performed in ImageJ (https://imagej.nih.gov/ij) [29], Microsoft Excel was used for data handling and Prism Graphpad version 8 (San Diego, CA, USA) was used for data presentation.

### 4.4. Two-Dimensional Fluorescence Difference Gel Electrophoresis

Whole cell proteomic analysis was performed by using 2-D fluorescence difference gel electrophoresis (DIGE) recommended by The Ohio State University Proteomics Shared Resource https://www.ccic.osu.edu/msp-proteomics [30]. U251 cells (1 × 10^7^) were incubated with 20 mM NaCl or 20 mM LiCl for 24 h. Cell pellets were snap frozen and processed for DIGE analysis by The Ohio State University Mass Spectrometry and Proteomics Facility using the Ettan DIGE system. After 2D-SDS-PAGE, gels were imaged with the Typhoon Variable Mode Imager. Image analysis and quantitation were performed using DeCyder Differential Analysis Software (GE Healthcare, Atlanta, GA, USA). Selected spots were excised and processed for protein/peptide identification.

### 4.5. Fluorescence Recovery After Photobleaching (FRAP) Assay

U251 cells were stably transfected with a pEGFP-vimentin expression vector, kindly provided by Professor Robert Goldman, Northwestern University [31]. High magnification, time-lapse image series of GFP-vimentin-labeled cells were collected using a Zeiss LSM510 confocal microscope system (Carl Zeiss Inc., Thornwood, NY, USA), equipped with an in vitro cell culture on-stage incubator (PeCon GmbH, Erbach, Germany). Images were collected every 10 s, for 20 min with minimal laser power (1%). A 5 μm^2^ area was burned with 100% laser power for 2 s (until the fluorescence from the selected area was equal to background levels). The half-time of fluorescence recovery T1/2 was analyzed using Zeiss LSM510 control software v3.2 (Jena, Germany). For image dynamic measurement 200-seconds-long, 50 frames sequences were collected. A frame-to-frame difference was calculated with ImageJ/Image Calculator function; data is presented as percent value of first frame of time sequence, accounting for background/noise. Co-localization of GSK-3 with vimentin was assayed on the same microscope system; U251 cells were transiently transfected with pcDNA4TO/CFP-hVimentin (NM_003380) and pcDNA4TO/YFP-GSK-3α/β plasmids. The pcDNA4TO plasmid was purchased from Thermo-Fisher/Life Technologies (cat# V1020), human vimentin was obtained from OriGene (SC111054), GSK-3α/β coding sequences were provided by Dr. Chris Phiel, Nationwide Children’s Hospital, Columbus, OH, USA.

### 4.6. GST Pull-Down Assay

U251 cells were transiently transfected with pDEST27/GST (control) and pDEST27/GST-GSK-3 (either α or β isoform) expression vector. A total of 500 μg of TX100 protein extract was incubated with 50 μL of glutathione agarose beads (Invitrogen, Carlsbad, CA, USA) at 4 °C for 1 h. Beads were extensively washed with TX100 lysis buffer and pulled-down proteins were extracted by boiling in 2× Laemmli Western blot sample buffer (Bio-Rad) and analyzed by Western blotting.

### 4.7. Digital Data Resources

Digital data presenting vimentin expression levels and Kaplan-Meier survival plots were obtained from The Cancer Genome Atlas (TCGA) (http://cancergenome.nih.gov/), and Rembrandt (https://caintegrator.nci.nih.gov/rembrandt/) databases.

## 5. Conclusions

Proteomic analysis of GBM cells treated with LiCl and other GSK-3 inhibitors showed alterations in multiple cytoskeletal proteins which may play a role in GBM migration. The intermediate filament protein vimentin was the most significantly altered of these, is highly expressed in GBM, and is prognostic, particularly in LGG. Knockdown of vimentin impairs GBM migration and its dynamics appear to be altered by LiCl treatment. Vimentin physically associates with GSK-3 and this may represent a novel mechanism that mediates intermediate filament dynamics and cell migration.

## Supplementary Materials

The following are available online at https://www.mdpi.com/2072-6694/11/4/466/s1, Figure S1: (a) The mRNA levels of vimentin collected from the TCGA database, Levels of transcript are presented as Fragments Per Kilobase of exon per Million reads (FPKM), Figure S2: The small subset of tissues for vimentin and labeled with DAB, Figure S3: LiCl and BIO Treated GBM cells reduces cell motility.
