# Supplementary material for: Proteomic Analysis Implicates Vimentin in Glioblastoma Cell Migration
Source: Cancers (Basel). 2019 Apr 3;11(4):466. doi: 10.3390/cancers11040466 (PMC6521049; doi:10.3390/cancers11040466)
Supplement: Supplementary file 1 [file cancers-11-00466-s001.pdf]

Supplementary Materials: Proteomic analysis implicates vimentin in glioblastoma cell migration

Michal O. Nowicki, Josie L. Hayes, E. Antonio Chiocca and Sean E. Lawler

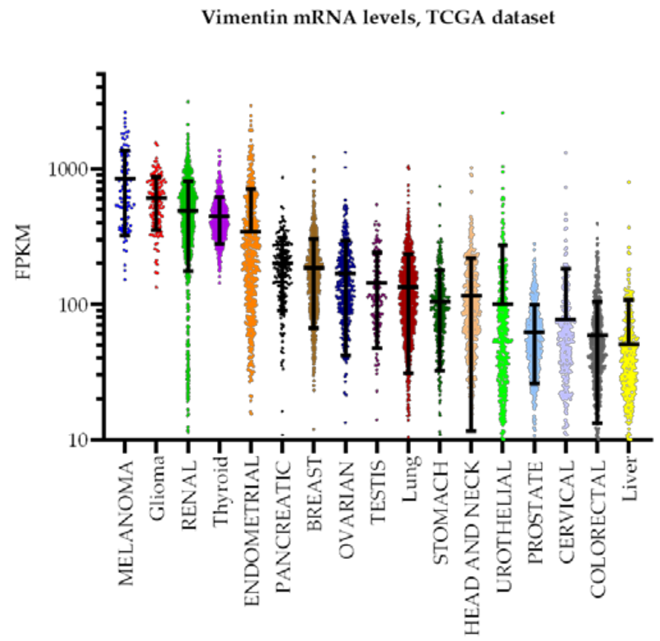

(**a**)

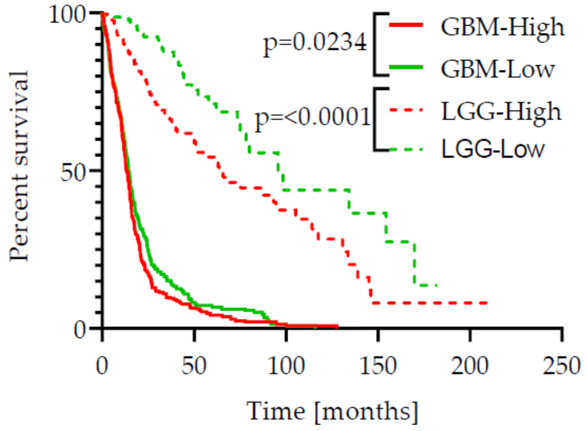

(**b**)

Figure S1. (**a**) The mRNA levels of vimentin collected from the TCGA database, Levels of transcript are presented as Fragments Per Kilobase of exon per Million reads (FPKM). (**b**) The Kaplan-Meier survival plots for TCGA datasets: GBM (combined platforms Affymetrix, Agilent and RNAseq) and combined data for Low Grade Gliomas (LGG).

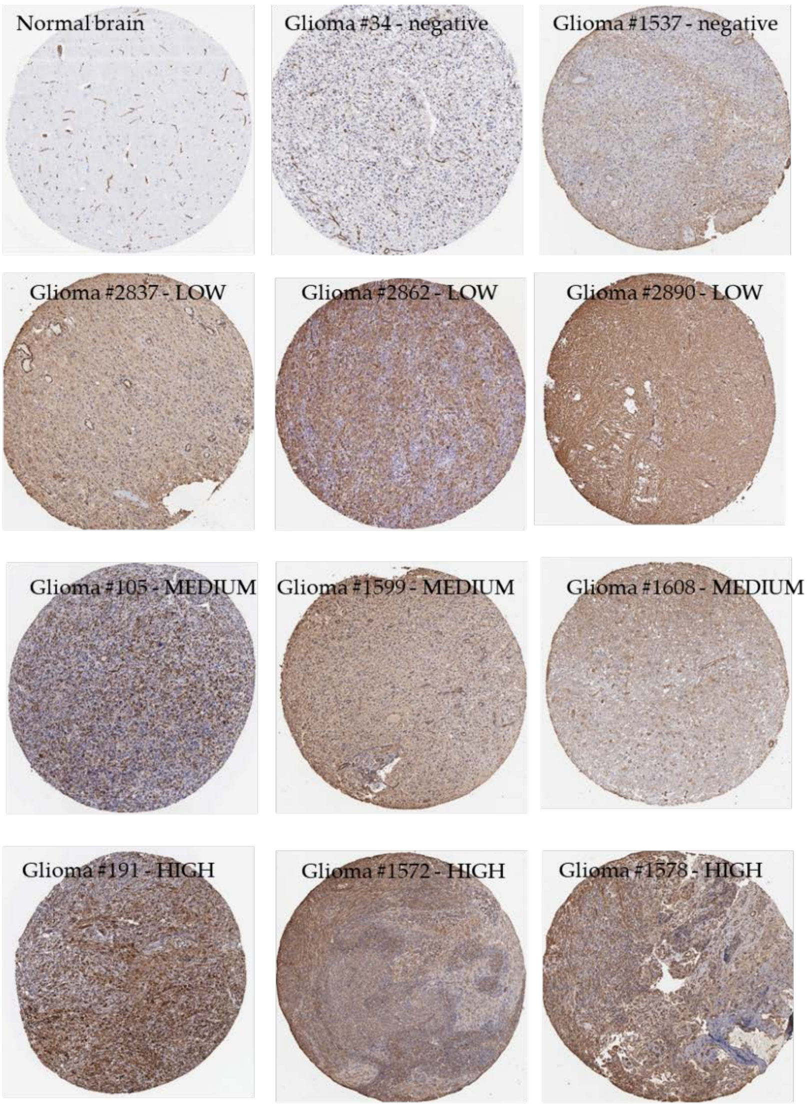

Figure S2. The small subset of tissues immunestained (Sigma cat# HPA001762) for vimentin and labeled with DAB (3,3′-diaminobenzidine). Data from The Human Protein Atlas [1], www.proteinatlas.org.

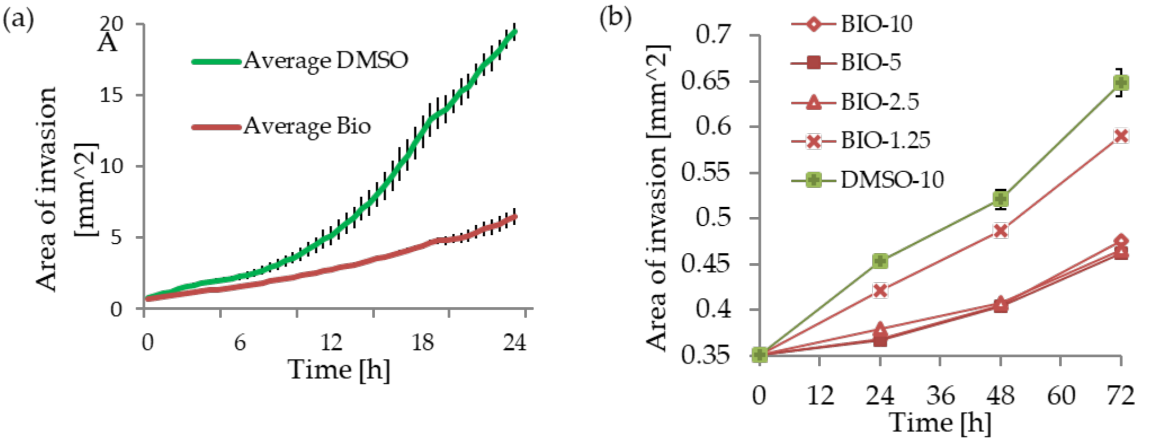

Figure S3. LiCl and BIO Treated GBM cells reduces cell motility. (**a**) Time lapse version of trans-membrane migration assay performed with FluorBlock inserts, U251pCDH cell line. (**b**) Spheroid migration invasion in collagen gel, G30pCDH.

References

1. Uhlén, M.; Fagerberg, L.; Hallström, B.M.; Lindskog, C.; Oksvold, P.; Mardinoglu, A.; Sivertsson, Å.; Kampf, C.; Sjöstedt, E.; Asplund, A.; et al. Proteomics. Tissue-based map of the human proteome. *Science* **2015**, *347*, 1260419.

© 2019 by the authors. Licensee MDPI, Basel, Switzerland. This article is an open access article distributed under the terms and conditions of the Creative Commons Attribution (CC BY) license (http://creativecommons.org/licenses/by/4.0/).
